# Failure of internal quality control in detecting significant reagent lot shift in serum creatinine measurement

**DOI:** 10.1002/jcla.22991

**Published:** 2019-08-02

**Authors:** Xiaoting Chen, Jia Wang, Wei Zhang, Erfu Xie, Bingfeng Zhang, Hua‐Guo Xu

**Affiliations:** ^1^ Department of Laboratory Medicine The First Affiliated Hospital of Nanjing Medical University Nanjing Jiangsu China

**Keywords:** average of normal, creatinine, quality control

## Abstract

**Background:**

Internal quality control (IQC) in clinical laboratories is carried out to monitor analytical stability. Usually, the satisfactory results of the IQC ensure the acceptability of the examination results. Here, we reported that patients' creatinine results are unreliable, although the internal quality control is satisfactory.

**Methods:**

Creatinine levels were analyzed from two quality control materials and twenty patients' specimens using two different lots of reagents. Lot‐to‐lot comparison was performed. The daily median values of serum creatinine levels of patients were calculated from the test results recorded in our laboratory information system.

**Results:**

Although IQC was consistent, serum creatinine concentrations were higher using lot B (median: 153 μmol/L; interquartile range: 122‐522 μmol/L) than using lot A (median: 133 μmol/L; interquartile range: 76‐508 μmol/L) for 20 patients (*P* = .001). The Deming linear regression showed a best fit of *y* = 0.9394 × *x* + 45.66. *R* ^2^ = .8919, and mean percentage difference between two lots was 34%. The new lot was considered unacceptable. Likewise, the median serum creatinine level from the 360 patients using lot B was 102 μmol/L, which was significantly higher than the daily medians of patients using lot A (median: 66 μmol/L; range: 61‐70 μmol/L) in the previous month.

**Conclusion:**

The variations in creatinine concentrations proved to be due to different lots of reagents. However, IQC materials tested using both lots of reagent exhibited minimal variation. Therefore, IQC alone is insufficient for assessing laboratory analytical results. This finding prompts us to be vigilant in potential pitfall of interpreting test results based on satisfactory IQC alone.

## 1 INTRODUCTION

Laboratory medicine plays an important role in clinical diagnosis, treatment, and monitoring. Laboratory test results influence medical decision‐making in two‐thirds to three‐quarters of cases.^1^ The goal of all clinical laboratorians is to provide high‐quality reported results in order to secure correct diagnosis, prediction, and decision‐making during treatment and follow‐up.^2^, ^3^, ^4^ The quality of results usually includes accuracy and reproducibility. The accuracy of laboratory tests is usually monitored through EQA (2‐3 times per year), while reproducibility of test results is commonly monitored through IQC. Internal quality control (IQC) represents an essential risk management tool within the total testing pathway (TTP) that contributes to the overall objective of assuring the quality of results produced in medical laboratories.^5^ IQC is primarily utilized in routine practice to monitor system performance (ie, make comparisons to what is expected under stable conditions), and allow analytical failures that affect performance to be detected.^5^ Reliable tests are contingent on passing both IQC and external quality assessment (EQA).

Here, we report an incident in which a batch of creatinine test results was found to be unreliable due to a change in reagent lots, even though IQC results were satisfactory.

## 2 MATERIAL AND METHODS

### 2.1 Experimental design

Twenty patients' serum specimens with serum creatinine concentrations that span the reportable range of the test method were obtained. The serum creatinine was tested with two reagent lots (hereinafter referred to as lot A and lot B). Samples were tested within 4 hours after collection and were stored at room temperature before tested. After each lot reagents had been changed, the creatinine was recalibrated and IQC was performed. Lot‐to‐lot differences in creatinine results were compared.

Consecutive patients' serum creatinine data from October 16 to November 15, 2017, were extracted from the Laboratory Information System (Neusoft), which were assayed using lot A. The median of the 360 patients' results that had been measured with the new lot on November 16 was calculated and compared with daily medians using lot A. All these data were from patients, including outpatient clinics and hospital wards.

### 2.2 Methods and equipment

Serum creatinine was measured by an enzymatic method on a Beckman Coulter AU5821 biochemical analyzer, using reagents and calibrators (lot 20170222 and lot 20170422) from Kehua Biological Products Co., Ltd, Shanghai. IQC materials were third‐party controls from Bio‐Rad Laboratories (lot 26400). To assess whether creatinine results are in control, the Westgard multiplication rules (1_3s_, 2_2s_, R_4s_, 4_1s_) are used.

### 2.3 Statistical analysis

Statistical analyses were performed with SPSS 19.0. The S‐W test was used to evaluate the normality of distribution. Wilcoxon signed‐rank test was used for significance testing between groups of continuous data (data are not normally distributed). *P* values <.05 were considered statistically significant.

Lot‐to‐lot comparison was performed with the Deming regression analysis for estimation of the slope and intercept. Difference between paired samples, and mean percentage difference between the results obtained with the two reagent lots were evaluated too. The acceptance criteria were slope between 0.90 and 1.10, intercept <6 μmol/L (<50% of lowest reportable value), *R* ^2^ > .95, and <10% mean difference between reagent lots.

## 3 RESULTS

### 3.1 Lot‐to‐lot differences

Serum creatinine levels of patients tested varied between the two reagent lots are shown in Table 1. The creatinine levels measured using two lots showed significantly different (*P* = .001). Although IQC was consistent and satisfactory, serum creatinine concentrations measured were higher using lot B (median: 153 μmol/L; interquartile range: 122‐522 μmol/L) than using lot A (median: 133 μmol/L; interquartile range: 76‐508 μmol/L) for 19 out of those 20 patients. According to the Analytical Quality Specification for Routine Analytes in Clinical Chemistry (WS/T 403‐2012, China) requirements, accepted total error for serum creatinine was 12%. Out of 20 patients, 10 were unacceptable (relative difference >12%). The difference and percentage difference decreased with increasing creatinine concentration measured (Table 1). The variation was remarkably higher (>10%, 10 of 11) for the specimens with creatinine concentration below 150 μmol/L.

**Table 1:** Lot‐to‐lot reagent differences for serum creatinine (unit: μmol/ L, rejection limit ± 12%)

Patients	A (A_R1_A_R2_ a)	B (B_R1_B_R2_ a)	B_R1_A_R2_ a	A_R1_B_R2_ a	Difference (B from A)	% Difference (B from A, %)	Result
1	43.5	94.8	98.4	46.2	51.3	117.9	Fail
2	51.7	121.1	125	55.2	69.4	134.2	Fail
3	57.1	90	91.6	60.1	32.9	57.6	Fail
4	66.6	145.1	146.3	69.4	78.5	117.9	Fail
5	76	106.9	108.1	77.2	30.9	40.7	Fail
6	76.6	99.9	103.8	80.2	23.3	30.4	Fail
7	105	131.7	134.3	106.8	26.7	25.4	Fail
8	108.2	126.1	129.1	110.8	17.9	16.5	Fail
9	122.7	135.6	137.5	128.8	12.9	10.5	Pass
10	132.1	237.3	246.8	122.1	105.2	79.6	Fail
11	134.3	142	146.9	139.4	7.7	5.7	Pass
12	151.3	160.1	166.3	156.9	8.8	5.8	Pass
13	267.6	334.1	337.8	264.4	66.5	24.9	Fail
14	350.1	386.3	386.9	359.4	36.2	10.3	Pass
15	428.3	439.3	444.8	441.4	11.0	2.6	Pass
16	534.7	550	551.9	550.8	15.3	2.9	Pass
17	645.7	605.8	644.2	656	−39.9	−6.2	Pass
18	654.9	663.6	664.3	680.4	8.7	1.3	Pass
19	778.7	781.6	795.9	798.4	2.9	0.4	Pass
20	887.9	892.1	904.3	918.6	4.2	0.5	Pass
QC							
QC1	135	134.6	142	137.6	−0.4	−0.3	
QC2	449.5	445	455	451.5	−4.5	−1.0	
Statistics of 20 patients' data							
Median	133.2	152.6	156.6	134.1			
Interquartile range	76.15‐508.10	122.35‐522.33	126.03‐525.13	77.95‐523.45			
*P* value^b^		0.001	0.000	0.002			

a Lot B_R1_A_R2_: Reagent 1 is lot B and Reagent 2 is lot A.

b Compared with lot A (A_R1_A_R2_), Wilcoxon signed‐rank test.

**Figure 1:**
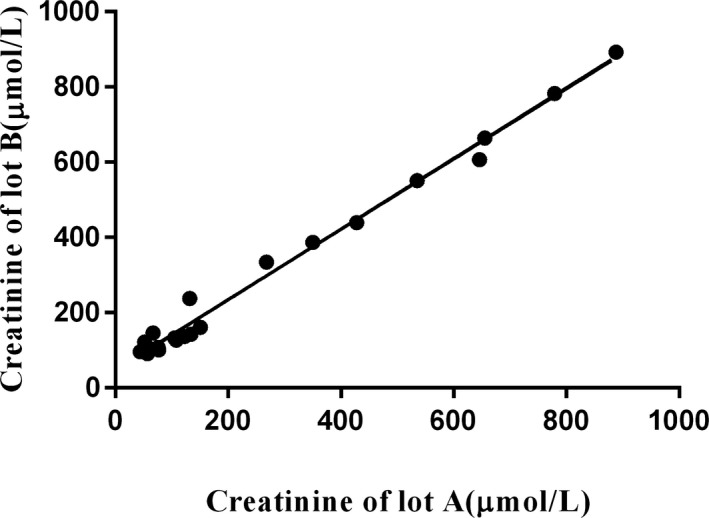
Scatter plot of creatinine results using lot A reagent compared with lot B. There is a correlation between the results of the two lots, *r* = .9444, *R* ^2^ = .8919, *P* < .0001. The Deming linear regression showed a best fit of *y* = 0.9394 × *x* + 45.66. According to acceptance criteria (slope between 0.90 and 1.10, intercept <6 μmol/L (<50% of lowest reportable value), *R* ^2^ > .95, and <10% mean difference between reagent lots), lot B was unacceptable

Figure 1 shows a linear regression analysis of the relationships between two lots. The correlation was 0.9444 and was statistically significant (*P* < .0001). The Deming linear regression showed a best fit of *y* = 0.9394 × *x* + 45.66, *R* ^2^ = .8919, and mean % difference was 34% (Table 1), so lot B was considered unacceptable on the basis of the predetermined criteria.

**Figure 2:**
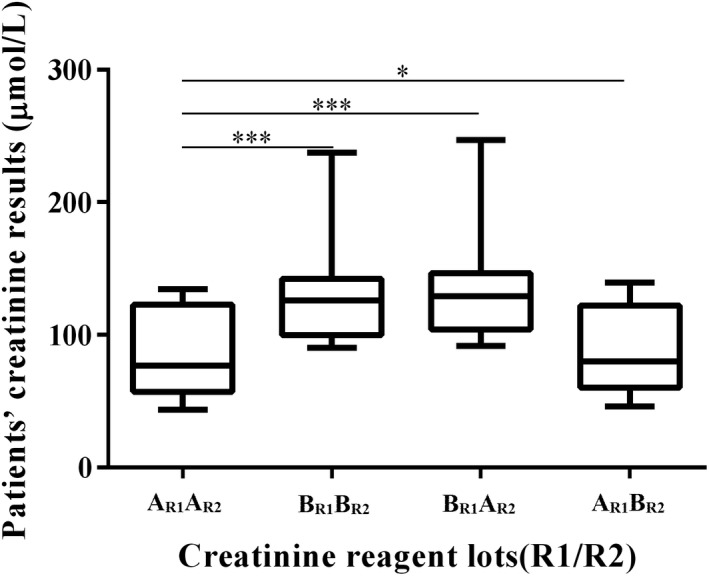
Lot‐to‐lot differences in creatinine reagent in patients' results less than 150 μmol/L. Line inside the box represents median, ends of the box represent interquartile range, and lines outside the box represent minimum and maximum. The patients' results of lot B_R1_B_R2_ (Reagent 1 and Reagent 2 were both B) and lot B_R1_A_R2_ (Reagent 1 was lot B and Reagent 2 was lot A) were all significantly higher than those of lot A_R1_A_R2_ (Reagent 1 and Reagent 2 were both A; both *P* values were 0.003). Triple asterisk indicates *P* < .01. The difference between lot A_R1_B_R2_ (Reagent 1 was lot A and Reagent 2 was lot B) and lot A_R1_A_R2_ was not statistically significant (*P* = .050). Asterisk indicates *P* ≥ .05

Figure 2 shows the difference in serum creatinine results below 150 μmol/L (lot A) measured using different lots of reagents. The results measured using lot B_R1_A_R2_ (Reagent 1 is lot B and Reagent 2 is lot A) and lot B_R1_B_R2_ were both obviously higher than those of lot A_R1_A_R2_ when creatinine was less than 150 μmol/L (both *P* values were .003). The difference in creatinine results measured between lot A_R1_B_R2_ and lot A_R1_A_R2_ was not statistically significant (*P* = .050). Nevertheless, the quality control results measured using lot B_R1_A_R2_, lot B_R1_B_R2,_ and lot A_R1_B_R2_ were satisfactory and showed no difference with using lot A_R1_A_R2_.

### 3.2 Medians of patients' data

**Figure 3:**
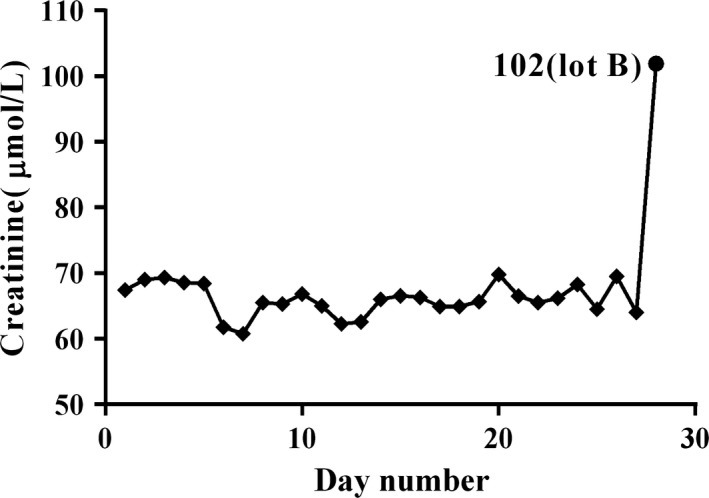
Comparison of the median of serum creatinine results using lot B reagent with the daily medians using lot A in the previous month. The daily medians using lot A reagent (square points) ranged from 61 μmol/L to 70 μmol/L. The median of the data measured with lot B reagent was 102 μmol/ L (dot point), which was significantly higher than the daily medians using lot A reagent

The daily medians of consecutive patients' creatinine results (analyzed using lot A) from October 16 to November 15, 2017, are shown in Figure 3. The median number of daily serum creatinine tests on the instrument was 714 (range: 298‐954). The median of daily medians was 66 μmol/L (range: 61‐70 μmol/L). Figure 3 shows that the median of the 360 patients' creatinine concentrations analyzed using lot B was 102 μmol/L (the last dot point in Figure 3), which was significantly higher than the daily medians of patients' results using lot A in the previous month.

## 4 DISCUSSION

We noticed on November 16, 2017, that laboratory tests on serum creatinine level of several patients did not match their clinical symptoms and deviate from their previous test results, although IQC had met the criteria in that run. And there were no changes in the procedure used on November 16 with reagent lot B. After reviewing the laboratory procedure and protocol, we found that the reagent for testing serum creatinine was accidentally changed to new lot (lot B) without verification on that day. The old lot had been used for more than 3 months successfully in our laboratory. Given the lot A also passed the proficiency test organized by National Center for Clinical Laboratories (NCCL, China) on September 2017 (PT score was 100%), it is plausible to speculate that the significant variation in test results may have resulted from the new lot. When replacing Reagent 2 of the lot A with Reagent 2 of the lot B, the deviation of the test results was negligible (Figure 2), demonstrating that Reagent 1 of the lot B skewed the test.

The change in reagent component materials, the instability, or deterioration of reagent composition during transportation or storage may cause new lot failure. The occurrence of noncommutable results for QC materials was frequent enough that the QC results could not be used to verify the consistency of results for patient samples when changing lots of reagents.^6^ The results showed that IQC worked as expected in spite of big variations in specimen test results between two lots of reagent. The verification of new reagent lot performance is not only a routine but also an important laboratory task.^7^ The Clinical and Laboratory Standards Institute (CLSI) EP26‐A guideline provides a lot‐to‐lot verification protocol to detect significant changes in test performance.^7^ Our laboratory's protocol for lot‐to‐lot verification consists of simultaneously testing five samples from both current and new lots. The relative difference for each sample is calculated. The new reagent lot is deemed acceptable only if the relative difference in at least four samples was less than the predefined rejection limit. Even so, verification of the new lot was overlooked by the technician and caused significant variations in test results. Fortunately, the issue was found before the patients' test reports were issued, and serum creatinine concentrations of these 360 patients were reanalyzed using the old reagent.

There are several reports on the use of patient results as a tool in monitoring analytical quality on the daily internal control.^3^, ^8^, ^9^, ^10^, ^11^, ^12^ We keep all laboratory data in our laboratory information system. Median value or average of normal (AON) can be easily calculated using statistical programs such as Excel, SPSS, or SAS. Any significant deviation from the median value or AON of previous test results can serve as an indicator for potential analytical error. IQC materials with a different matrix from clinical specimens may be insensitive to reagent changes, as observed here. Clinical specimens contain no artificial components and therefore are a useful component in assuring analytical performance. Moreover, all patients' results can be extracted from the laboratory information system without involving extra cost and labor as compared to the preparation of IQC materials.^3^ So, median value or AON can be used as another aspect of QA. In this study, with the reagent problems, the daily median of patient outcomes had changed significantly while IQC results were satisfactory, which suggested that the daily median could be a good complement to IQC.

## 5 CONCLUSIONS

In summary, our findings indicate that satisfactory IQC does not necessarily mean reliable analytical results in clinical laboratories. However, in practice, many laboratory staff heavily rely on IQC to assess the reliability of their test results. As reported here, some laboratory testing errors could not be revealed only through IQC and EQA, which could negatively impact on clinical diagnosis and treatment. To ensure the reliability of test results, reagent verification and analysis of patient previous test records should be implemented besides IQC and EQA.

## AUTHOR CONTRIBUTIONS

All the authors have accepted responsibility for the entire content of this submitted manuscript and approved the submission.

## ETHICAL APPROVAL AND PATIENT CONSENT

Ethical clearance for this study was obtained from the Ethics Committee at the First Affiliated Hospital of Nanjing Medical University. Because all the samples used in this study were collected from clinical residual specimen, written informed content from each patient was waived.
